# Evaluating the temporal and spatio-temporal niche partitioning between carnivores by different analytical method in northeastern Japan

**DOI:** 10.1038/s41598-022-16020-w

**Published:** 2022-07-14

**Authors:** Ryoga Watabe, Hiroshi Tsunoda, Masayuki U. Saito

**Affiliations:** 1 Graduate School of Agricultural Sciences, Yamagata University, 1-23, Wakaba-machi, Tsuruoka-shi, Yamagata, 997-8555 Japan; 2 Center for Environmental Science in Saitama, 914, Kamitanadare, Kazo-shi, Saitama, 347-0115 Japan; 3 Faculty of Agriculture, Yamagata University, 1-23, Wakaba-machi, Tsuruoka-shi, Yamagata, 997-8555 Japan

**Keywords:** Community ecology, Animal behaviour, Ecology, Zoology

## Abstract

Temporal and spatio-temporal niche partitioning is an important strategy for carnivore coexistence. Camera-trap data has been analyzed through several methods to assess the temporal and spatio-temporal niche partitioning. However, different analytical approaches used to may evaluate niche partitioning detect different results. In this study, we evaluated the temporal or spatio-temporal partitioning among sympatric medium-sized carnivores, red foxes, raccoon dogs, and Japanese martens, based on three analytical methods—the temporal overlap, temporal co-occurrence, and time-to-encounter analysis—to evaluate. From May to October 2019 and 2020, we obtained the activity of the target species using camera-traps in northeastern Japan. We analyzed the data with the coefficient of temporal overlap, probabilistic co-occurrence analysis, checkerboard score, and multi-response permutation procedures. The results of the assessment of the niche partitioning differed depending on the analytical methods based on temporal and spatio-temporal partitioning. Therefore, we conclude that the choice of analytical approach is important for evaluating the temporal and spatio-temporal niche partitioning.

## Introduction

The competitive exclusion principle states that two ecologically similar species cannot coexist^1^. Thus, multiple sympatric species can partition their niche according to major factors: food resources, space, and time^2^. Elucidating the mechanisms of species coexistence based on their niche partitioning is important for understanding community diversity and assemblage, and for implementing effective ecosystem conservation and management strategies^3–6^.

It is widely known that interspecific competition can often occur among sympatric carnivores^7–10^. The extent of their interspecific competition is influenced by taxonomic similarity, dietary overlap, and intermediate body-size differences^11^. For example, larger coyotes (*Canis latrans*) can exclude smaller swift foxes (*Vulpes velox*) from their home ranges and territories^9^. Tsunoda et al.^12^ suggested that interspecific competition can be high between larger golden jackals (*Canis aureus*) and smaller red foxes (*Vulpes vulpes*) because of their dietary overlap. To avoid such competition, carnivores coexist sympatrically by shifting their niches (e.g.,^13–15^). Temporal niche partitioning is one of the important strategies to ensure their coexistence^5,16,17^.

Temporal niche partitioning among sympatric carnivores is assessed in several methodologies, such as camera-trappping^5^ and radio-telemetry^10^. Currently, that is often assessed using camera-trap data^5^, and several studies have demonstrated that temporal niche partitioning is can be a factor for their successful sympatry (e.g.,^18–21^). For example, European badgers (*Meles meles*) and stone martens (*Martes foina*) shifted their diel activity patterns to avoid antagonistic encounters with larger golden jackals in Bulgaria^20^. Stone martens also tended to be active at different times than larger red foxes and European wildcats (*Felis sylvestris*)^21^. Typically, temporal niche partitioning among carnivores is assessed using time data (i.e., 0:00–23:59) (e.g.,^18–23^). Among the assessments using time data, the coefficient of temporal overlap of activity patterns based on the kernel density estimation^24,25^ has been widely used (e.g.,^5,26–30^). This coefficient is used to assess temporal niche partitioning in terms of the degree of overlap in diel activity patterns between species^25^. Frey et al.^5^ argued that the kernel density estimation has greatly improved the level of knowledge available from camera-trap data.

Furthermore, recent studies have assessed the influence of effective sample size for the accuracy and the statistical power when estimating the temporal overlap^31,32^. However, measuring the temporal overlap may sometimes be insufficient to assess species interactions correctly, as this method evaluates the overlaps/differences in diel activity patterns between a focal species pair throughout the day, from 0:00 to 23:59, from a dataset pooled during a sampled period. For example, even if two species are both nocturnal and their diel activity patterns are overlapped, a subordinate (i.e., smaller) species may spatio-temporally avoid direct encounters with the larger competitor based on detecting the competitor at fine time scale^33^. Nowadays, the spatio-temporal niche partitioning has been measured to assess behavioral avoidance by focusing on the time-to-encounter between individuals of different species (e.g.,^33–39^). Indeed, Karanth et al.^33^ found a large overlap in diel activity patterns between tigers (*Panthera tigris*) and leopards (*Panthera pardus*), while demonstrating their behavioral avoidance by using the time-to-encounter analysis. Similarly, Paúl et al.^38^ also found a large overlap in diel activity patterns between side-striped jackals (*Canis adustus*) and African wolves (*Canis lupaster*), while indicating the occurrence of some behavioral avoidance using the time-to-encounter analysis, with side-striped jackals taking longer than expected to be detected after the occurrence of African wolves. These results suggest that the evaluation of behavioral avoidance at fine time scales using the time-to-encounter analysis (i.e., evaluation of spatio-temporal partitioning) may provide an understanding of the mechanisms of species coexistence that cannot be detected by only estimating the temporal overlap (i.e., evaluation of temporal partitioning). When we use such analytical approaches focusing different scales, we will have a better understanding mechanisms of species coexistence.

**Figure 1:**
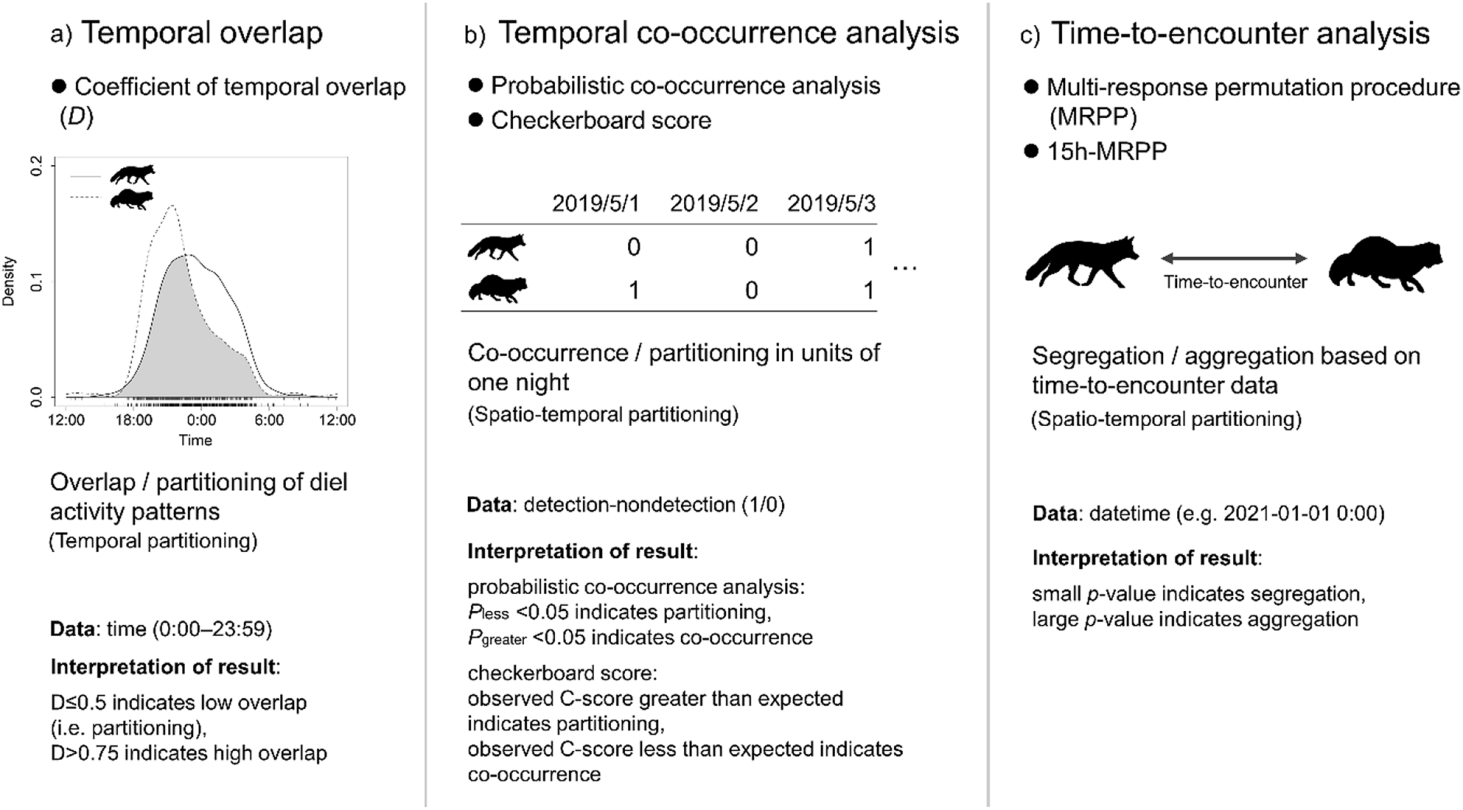
Images illustration and comparison of the different analytical approaches used in this study. The interpretation of results were referred from Monterroso et al*.*^19^ for coefficient of temporal overlap, Griffith et al*.*^41^ for probabilistic co-occurrence analysis, McCreadie et al*.*^71^ for checkerboard score, Karanth et al*.*^33^ for time-to-encounter analysis. The drawing of the kernel density estimation in panel (**a**) is based on the data from this study (see Fig. 3). The silhouette images of the red fox and raccoon dog were public domain data obtained from PhyloPic (http://phylopic.org/).

We evaluated the temporal and spatio-temporal partitioning among carnivores based on multiple analytical methods. We used the temporal overlap, temporal co-occurrence analysis, and the time-to-encounter analysis (Fig. 1). Since the temporal overlap (e.g.,^5,26–30^) and time-to-encounter analysis (e.g.,^33,36–38^) has been often used in the evaluation of temporal partitioning and spatio-temporal partitioning respectively, we chose these methods for our comparison. In this study, we also applied the spatial co-occurrence analysis^40,41^, which is a method used to assess the spatial co-occurrence of multiple species by using presence-absence data (e.g.,^42–45^), to the temporal co-occurrence analysis (Fig. 1b). We considered that this analysis can evaluate the spatio-temporal responses of carnivores focusing on the date rather than the time (i.e., 0:00–23:59) using detection-nondetection data obtained by camera-trapping (for more details see ‘Methods’). The temporal co-occurrence analyses might have methodological advantages, due to use of presence-absence data. Presence-absence data can be obtained by other survey methods besides camera traps, such as field signs and acoustic monitoring, and are typically available in large amounts of data. It would be useful to examine whether spatio-temporal niche partitioning can be evaluated based on presence-absence data.

Our focal species were red foxes, raccoon dogs (*Nyctereutes procyonoides*), and Japanese martens (*Martes melampus*). Red foxes and genus *Martes* individuals are widespread in the Northern Hemisphere^46,47^, and raccoon dogs are widely distributed in East Asia and Russian Far East and have also been introduced in Europe^48^. The diet of the focal three species is generally overlapped^49,50^. In addition, the red foxes and raccoon dogs belong to the same family (i.e., Canidae), and differ in body size from the Japanese martens^47^, indicating their potential competitive interactions, according to Donadio and Buskirk^11^. In Europe and North America, several studies have reported that red foxes killed genus *Martes*^13,51,52^. Therefore, our focal species are ideal to assess the role of temporal niche partitioning in carnivores sympatry.

## Methods

### Study area

**Figure 2:**
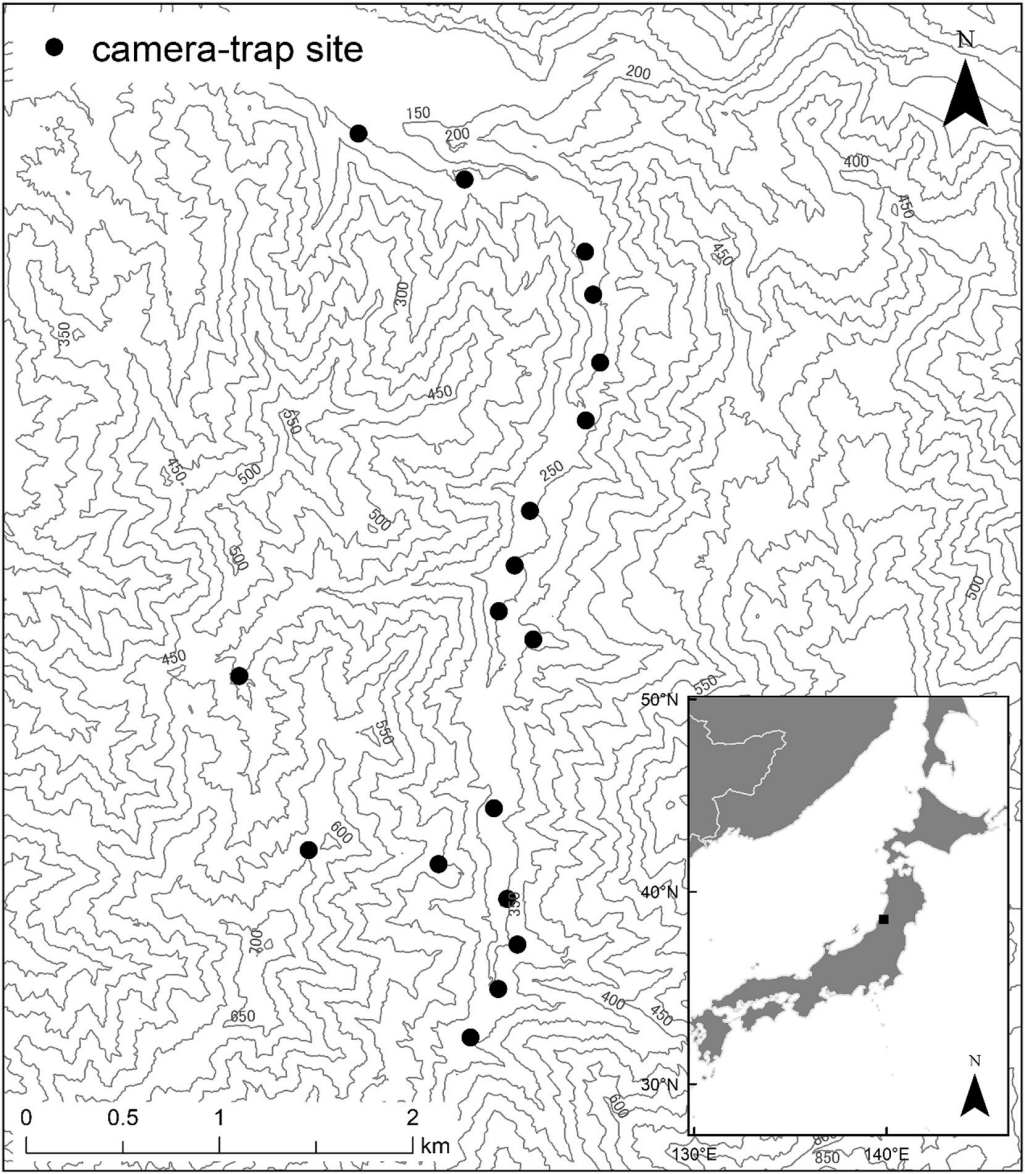
Study area and locations of camera-trap sites. This map was created using ArcGIS 10.7 (https://www.esrij.com/products/arcgis/). Borders were drawn using data from World Borders Dataset by thematicmapping.org (http://thematicmapping.org/). Contour lines were created using a 5 m digital elevation model from Fundamental Geospatial Data by Geospatial Information Authority of Japan (https://www.gsi.go.jp/kiban/index.html).

We collected animal images by camera-trapping in the Experimental Forest of Yamagata University in Yamagata Prefecture, northeastern Japan, belonging to a cool temperate climatic zone (38°33′N, 139°51′E; Fig. 2). The three target species in this study were confirmed to be sympatric in this area^30^. The annual mean temperature is approximately 10.5 °C, and the annual precipitation is approximately 3,300 mm (average from 2019 to 2020). The altitude ranges from approximately 230 to 850 m. The study area has heavy snowfall, with the maximum snow depth exceeding 3 m in winter. Apart from the three focal mesocarnivores in this study, seven other medium- and large-sized mammals were present in the study area: the Japanese hare (*Lepus brachyurus*), Japanese macaque (*Macaca fuscata*), masked palm civet (*Paguma larvata*), Japanese badger (*Meles anakuma*), Asiatic black bear (*Ursus thibetanus*), Japanese weasel (*Mustela itatsi*), and Japanese serow (*Capricornis crispus*)^53^. The major canopy species are beeches (*Fagus crenata*), oaks (*Quercus crispula*), maples (*Aceraceae*), and cedar (*Cryptomeria japonica*) plantations.

### Camera trapping

Between May 1 and October 31, 2019 and 2020, we set 18 camera-trap sites using infrared-triggered cameras (models #BTC-6HD-940, Browning, AL, USA and #BTC-6HD-APX, Browning, AL, USA) (Fig. 2; Supplementary Table S1). Previous studies have reported that the effective detection distance of infrared-triggered cameras is shorter with higher vegetation density^54^ and that the detection rate of cameras is higher on forest roads than in forests^55^. To mitigate the change in the detection distance due to the increase in vegetation density, and because the detection rates of the three focal species were also higher on the forest road than forest interior in this study area^53^, we installed all cameras on trees along to a forest road, approximately 1.5 m above the ground. We did not use baits or lures. We programmed the cameras to capture three images for each trigger, with a 1 min delay between each trigger. We identified the focal species from the three images and recorded the species, camera-trap site, date, and time as a single detection data.

### Data analysis

#### Spatial autocorrelation

Positive spatial autocorrelation can arise because of repeated detections of the species and can affect the assessment of species interactions when cameras are set close together within a small area^21^. To assess the positive spatial autocorrelation, we used the Mantel’s correlogram^56^ before analyzing the temporal niche partitioning. Correlation indices were calculated using 10,000 randomizing simulations based on the number of detections of each target species per day per camera and the longitudinal/latitudinal positions of cameras. According to Tsunoda et al.^21^, when we collected consecutive detections of the same species at a camera-trap site within 30 min, we treated it as a single sample. This analysis was performed using the ‘vegan’ package^57^ in statistical software R^58^ ver. 3.5.2.

#### Temporal overlap

We first estimated the diel activity patterns of target species as a probability density function using the kernel density estimation^24^. To determine the interspecific temporal overlap (Fig. 1a), we estimated the coefficient of temporal overlap (*D*) for each species-pair, which ranged from 0 (no overlap) to 1 (complete overlap)^24,25^. This is defined as the area under the curve that is formed by taking the minimum of the two density functions at each time point^59^. Since the coefficient reliability depend on the sample size^25^, we followed the criteria for obtaining reliable estimates proposed by Meredith & Ridout^25^. According to Meredith and Ridout^25^, we used the *D*_4_ method because each focal species dataset contained more than 75 samples. We defined the *D* ≤ 0.5 as “low”, 0.5 < *D* ≤ 0.75 as “moderate”, and *D* > 0.75 as “high”, according to Monterroso et al.^19^. To assess the reliability of the *D* statistic and its 95% confidence intervals (*CI*), we performed a smoothed bootstrap with 10,000 bootstrap samples^25^. To reduce duplicate counts of the same individual, when we collected consecutive detections of the same species at a camera-trap site within 30 min, we treated it as a single sample (e.g.,^19,21,28,30,60^). These analyses were performed using the ‘overlap’ package^25^ in R.

#### Temporal co-occurrence analysis

To assess whether the three target species avoid each other in units of one night, we performed the temporal co-occurrence analysis using a matrix containing the detection-nondetection (1/0) data per night at each camera site for each species pair (Fig. 1b). We only analyzed nighttime because the three target species were nocturnal in this study area^30,60^. We used two methods: the probabilistic co-occurrence analysis^41^ and the checkerboard score^40^. Both methods determine the probability that the observed frequency of co-occurrence of two species is less than, greater than, or not different from the expected frequency, if the two species occurred independently from each other in units of one night. In the probabilistic co-occurrence analysis, first, the observed co-occurrence rate for each species was calculated by dividing the number of detections (1) for one species by the total number of nights at each site. Second, the expected co-occurrence was calculated by multiplying the observed co-occurrence rate of one species, the observed co-occurrence rate of other one species, and the total number of nights at each site^41^. Finally, we compared this calculated expected co-occurrence to the number of observed co-occurrence^41^. In the checkerboard score, we created a null model for comparison to evaluate co-occurrence patterns in observed matrix, by repeating the process of randomly sorting the 0/1 data in each row of the observed matrix 1000 times, without changing the overall number of detections. The null model and observed matrix were used for the degree of species co-occurrence, expressed as the C-score statistic^40,43^. We defined one night as 60 min before sunset to 60 min after sunrise. We obtained the sunrise and sunset times for each survey day by using the R package ‘rSetDayNightAttr’^61^. The analyses were performed using the ‘cooccur’ and ‘vegan’ packages^41,57^ in R.

#### Time-to-encounter analysis

To assess the behavioral avoidance between different species by using the time-to-encounter analysis (Fig. 1c), we used multi-response permutation procedures^62^ (hereafter, MRPP) according to Karanth et al.^33^. To determine competitive dominances among the three target species, we considered the before/after occurrence (e.g. red fox (before)—raccoon dog (after), raccoon dog—red fox) for the detected species when calculating the time-to-encounter across each species pair^38^. For this procedure, we created matrix of detection records for each species consisting of the camera-trap site, date, and time. For every detection record, we calculated the minimum time to the subsequent detection between species pairs for each camera-trap site in each survey duration (seven sites: May 1–Oct 31, 2019, four sites: Aug 19–Oct 31, 2019, four sites: May 1–Oct 31, 2020, eight sites: Aug 24–Oct 31, 2020). Thus, for each species pair, we obtained a set of observed times-to-encounter. To compare this to a random expectation (i.e., a null model representative of neither segregation nor aggregation), we randomly permutated the detection records of subsequent species among camera-trap sites with the same survey duration. We used this random permutation to re-calculate the time-to-encounter and repeated this process 1000 times^33^. Finally, we compared the observed median time-to-encounter and the 1000 medians of the random permutations. We finally calculated the *p*-values as the proportion of times the observed median was larger than the medians of the random permutations^33^. A large *p*-value indicates spatio-temporal aggregation (i.e., the observed time-to-encounter was shorter than the random expectation) while a small *p*-value indicates spatio-temporal segregation^33^. In the previous studies that have conducted time-to-encounter analysis (e.g.,^33,35^), consecutive detections of the same species less than 1 min apart were collapsed into a single sample. In this study, we set the cameras’ interval to 1 min, therefore we used all detection data for this analysis. We used statistical software R for this analysis.

In the MRPP, the time-to-encounter data between two species may span multiple days^33^. However, our focal species are typically nocturnal^30,60^ and the time-to-encounter data may include daytime when they are inactive or at the resting sites. Considering these factors, we performed the MRPP with an upper limit on the time-to-encounter data to focus on a more detailed scale. To focus on the nocturnal activity time of the target species, we used only the time-to-encounter data within 15 h, and assessed the behavioral avoidance between species using the same calculation method and evaluation criteria in the MRPP described above (hereafter, 15 h-MRPP). We defined the time-to-encounter within 15 h as the longest nighttime during the survey period (13 h) with one hour before sunrise and after sunset.

## Results

The total number of camera-days at all camera-trap sites was 2,826, excluding the number of days when the angle of the cameras was changed because of bear attacks or when technical issues occurred. We obtained 705, 735, and 289 detection data of red foxes, raccoon dogs, and Japanese martens, respectively.

We found no positive spatial autocorrelation in the first and second proximal distance classes (Supplementary Fig. S1). This result indicated that proximity between cameras did not affect spatial similarities in detected species. Additionally, the relative detection frequency was not similar among the camera-trap sites during the same survey period (Supplementary Fig. S2), indicating the species did not often keep traveling along the forest road. Therefore, we assumed that the proximal camera-trap placement on the forest road had little effect on the assessment of species interactions.

### Analysis of temporal overlap

**Figure 3:**
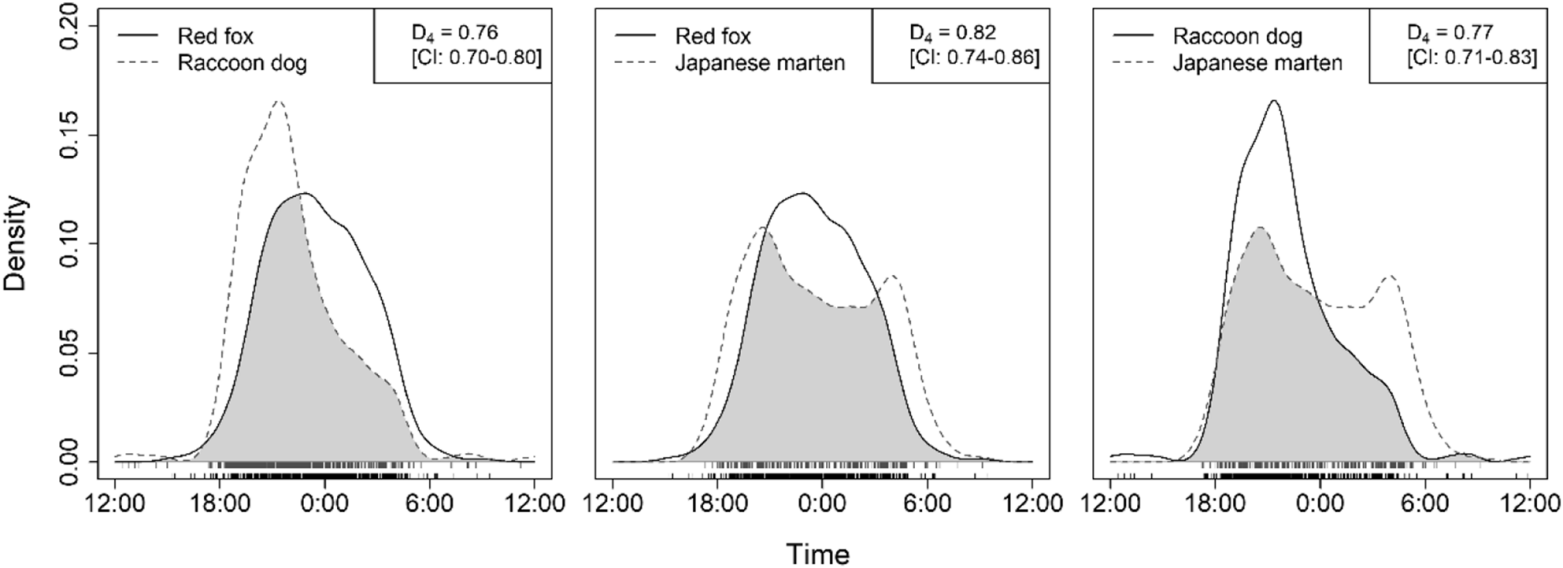
Temporal overlap between species. The gray area indicates the overlap between diel activity patterns. The rug at the bottom of the plot indicates the original observations of activity time. The number of samples used for the kernel density estimation was 669 for red foxes, 691 for raccoon dogs, and 282 for Japanese martens.

The *D* was higher than 0.75 for all species pairs [red fox–raccoon dog was 0.76 (CI: 0.70–0.80), red fox–Japanese marten was 0.82 (CI: 0.74–0.86), and raccoon dog–Japanese marten was 0.77 (CI: 0.71–0.83)] (Fig. 3). Therefore, the diel activity patterns were largely overlapped among the species.

### Temporal co-occurrence analysis

The results of the probabilistic co-occurrence analysis showed that the observed frequency of co-occurrence of red foxes and raccoon dogs and of raccoon dogs and Japanese martens was greater than the expected frequency (Table 1). This indicates that these species pairs significantly co-occurred temporally. The observed frequency of co-occurrence of red foxes and Japanese martens was not different from the expected frequency (Table 1).

**Table 1:** Results of the probabilistic co-occurrence analysis.

Species	Observed cooccurrence	Expected cooccurrence	*P*_less_	*P*_greater_
Red fox and Raccoon dog	96	83.0	0.962	0.049*
Red fox and Japanese marten	40	43.1	0.326	0.736
Raccoon dog and Japanese marten	55	41.4	0.993	0.011*

Observed cooccurrence is the number of observations in which two species cooccur during one night. Expected cooccurrence represents the expected frequency of two species co-occurring during one night. *P*_less_ represents the probability that the two species would co-occur at a frequency smaller than the observed frequency, if the two species had occurred randomly (independently). *P*_greater_ represents the probability that the two species would co-occur at a frequency greater than the observed frequency, if the two species had occurred randomly (independently). The number of presence of data was 479 for red foxes, 460 for raccoon dogs, and 239 for Japanese martens. Asterisk represents *P* < 0.05.

The results of the checkerboard score showed that the observed frequency of co-occurrence of raccoon dogs and Japanese martens was greater than expected frequency (Table 2), indicating that this species pair significantly co-occurred temporally. The observed frequency of co-occurrence of red foxes and raccoon dogs and of red foxes and Japanese martens was not different from the expected frequencies (Table 2).

**Table 2:** Results of the checkerboard score. Simulated C-score represents the mean and confidence interval of a null model distribution generated using a randomization algorithm.

Species	Observed C-score	Simulated C-score	SES	*P*
Red fox and Raccoon dog	0.040	0.042 (CI 0.039–0.046)	− 1.698	0.102
Red fox and Japanese marten	0.025	0.024 (CI 0.022–0.026)	0.547	0.653
Raccoon dog and Japanese marten	0.021	0.023 (CI 0.022–0.025)	− 2.359	0.023*

The positive SES (standardized effect size) indicates an observed co-occurrence at a rate smaller than that expected by chance (partitioning); a negative effect size indicates an observed co-occurrence at a rate greater than that expected by chance (co-occurrence). The number of presence of data was 479 for red foxes, 460 for raccoon dogs, and 239 for Japanese martens. Asterisk represents *P* < 0.05.

### Time-to-encounter analysis

**Figure 4:**
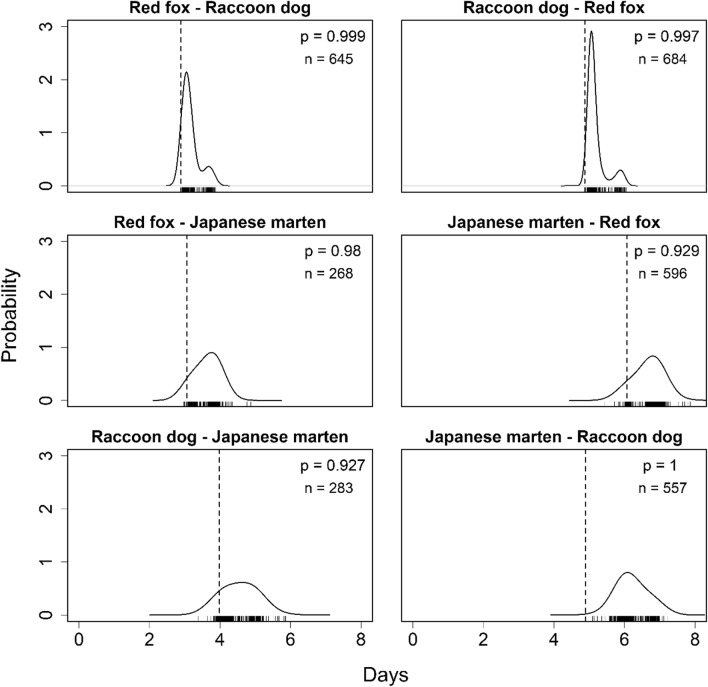
Results of the time-to-encounter analysis using the MRPP. Red fox-raccoon dog represents that raccoon dogs was detected after red foxes, for example. The dashed vertical lines represent the observed median time-to-encounter between two species. The rug plot represents the 1,000 medians of the random permutations and the curve represents the distribution estimated from them; n represents the number of the observed time-to-encounter. The *p*-values indicate the proportion of times the observed median was larger than the medians of the random permutations.

As a result of the MRPP, the *p*-values for all species pairs were greater than 0.92, indicating their spatio-temporal aggregations (Fig. 4).

**Figure 5:**
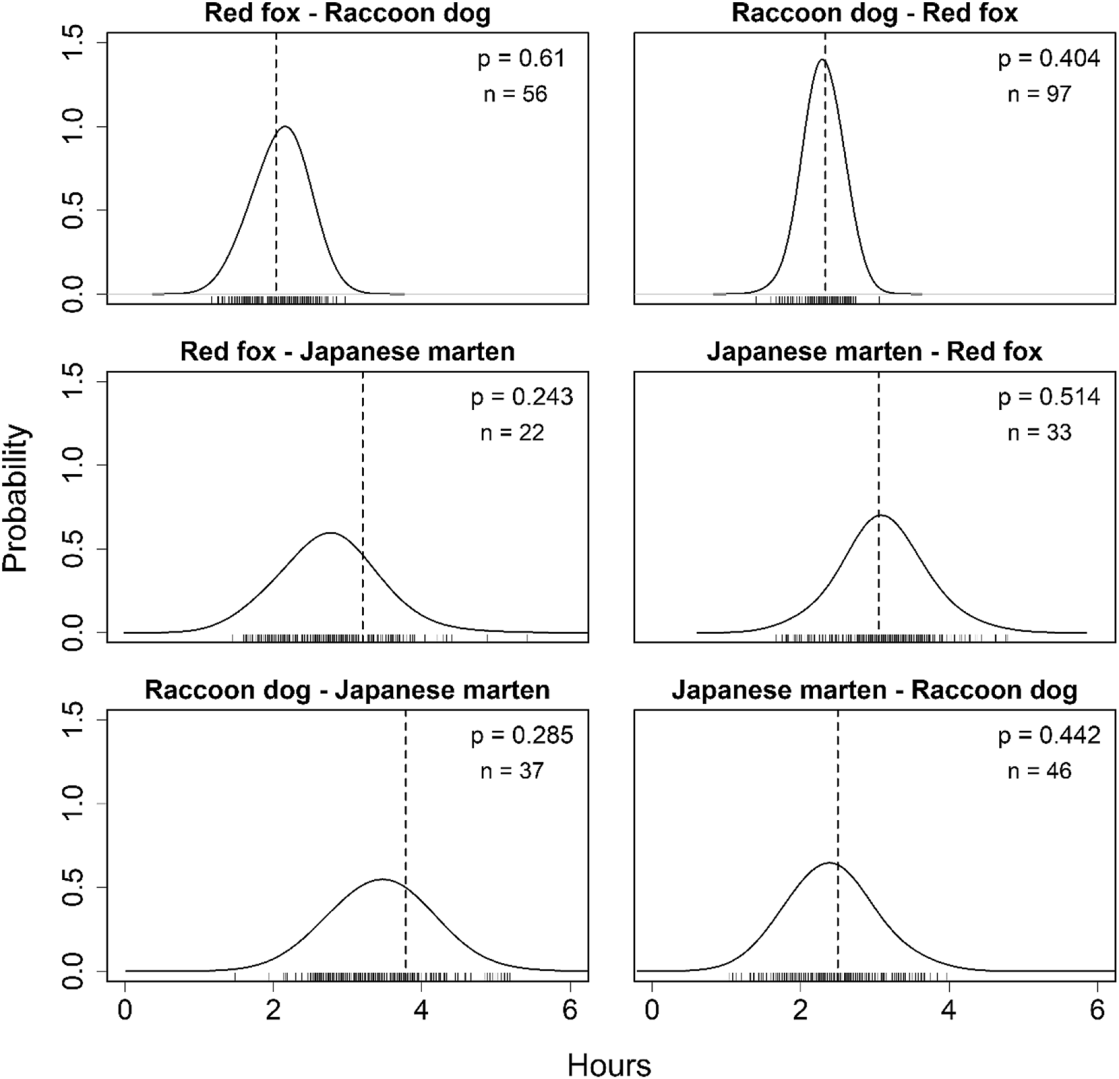
Results of the time-to-encounter analysis using the 15-MRPP. Red fox-raccoon dog represents that raccoon dogs was detected after red foxes, for example. The dashed vertical lines represent the observed median time-to-encounter between two species. The rug plot represents the 1,000 medians of the random permutations and the curve represents the distribution estimated from them; n represents the number of the observed time-to-encounter. The *p*-values indicate the proportion of times the observed median was larger than the medians of the random permutations.

The results of the 15 h-MRPP showed that the observed median time-to-encounter and the medians of the random permutations did not differ substantially for all species pairs (Fig. 5). Although strong evidence for spatio-temporal segregations among species was not detected, the *p*-values for the red fox – Japanese marten and raccoon dog – Japanese marten pairs were smaller than those of the other pairs, which were closer to the segregation values than aggregation (*p* = 0.243, *p* = 0.285, Fig. 5).

## Discussion

The degree of temporal partitioning differed among the compared analytical methods, which use temporal data differently (Table 3). Although the coefficients of temporal overlap (*D*) indicated large overlaps in diel activities among all species pairs (Fig. 3), the temporal co-occurrence analyses did not detect co-occurrence nor partitioning between red foxes and Japanese martens (Tables 1 and 2). Further, because of the time-to-encounter analysis, while the MRPP showed spatio-temporal aggregations among all species pairs (Fig. 4), the 15 h-MRPP detected marginal segregations in the pairs where martens were detected after the other species (Fig. 5). The coefficient temporal overlap evaluates the overlaps/differences in diel activity patterns between a focal species pair throughout the day, from 0:00 to 23:59, from a dataset pooled during a sampled period. The MRPP evaluates time-to-encounter data with multiple temporal scales that from several hours (i.e., within a night) to several days or weeks (i.e., multiple active and inactive periods). These methods potentially overestimated the temporal niche overlaps, by evaluating longer time periods than their “real” active time. In contrast, both the temporal co-occurrence analysis and the 15 h-MRPP evaluated only nighttime data that were consistent with our focal species’ activity patterns. Our results suggest that only using the coefficient of temporal overlap or the MRPP with multiple-day data was insufficient to accurately determine temporal niche partitioning, specifically when both species show similar activity patterns, such as our focal species. Moreover, the 15 h-MRPP evaluates the behavioral avoidances using time-to-encounter data that focused on a finer temporal scale (several hours in a night) than those of the other analytical methods, differentiating the results of temporal niche partitioning from the other analytical methods. Our findings indicated that the evaluation of temporal niche partitioning from multiple analytical methods with different temporal-scale data is necessary.

**Table 3:** Comparison of all analysis results.

Species	Temporal overlap	Probabilistic co-occurrence analysis	Checkerboard score	MRPP	15 h-MRPP
**Red fox and Raccoon dog**					
Red fox–Raccoon dog	+	+		+	
Raccoon dog–Red fox				+	
**Red fox and Japanese marten**					
Red fox–Japanese marten	+			+	–
Japanese marten–Red fox				+	
**Raccoon dog and Japanese marten**					
Raccoon dog–Japanese marten	+	+	+	+	–
Japanese marten–Raccoon dog				+	

+ represents a result closer to the overlapping side of the temporal niche and – represents a result closer to the partitioning side of the temporal niche.

Although we did not identify which factors were involved, there are several possibilities for the temporal niche overlaps between the target species in this study (Table 3). First, predators typically synchronize their activities with those of their prey, which results in similar activity patterns among predators using the same prey^63,64^. In Japan, the sympatric red foxes, raccoon dogs, and Japanese martens commonly prey on small mammals^49^. Therefore, the activity patterns of our focal species might be similar due to their synchronization with those of their staple prey species. Second, the timing of the activities of multiple species may coincide with each other due to weather conditions. Hendrichsen & Tyler^65^ indicated that meteorological factors such as temperature, precipitation, and wind can affect rates of heat loss for wild animals, affecting their activity patterns and narrowing the temporal niche partitioning between species^66^. The results of the 15-MRPP indicated the spatio-temporal avoidance by martens against the other two species, though the statistical evidence was marginal (*p* = 0.243, *p* = 0.285, Fig. 5). On a more detailed temporal scale, olfactory cues from competitive species provoke behavioral avoidance^67^. Barrull et al.^68^ demonstrated that the stone martens rarely appeared within 60 min of the detection of the red foxes and European badgers (*Meles meles*), indicating that smaller martens may detect odors from the larger competitors and avoid them temporally. The Japanese marten is the smallest among our focal mesocarnivores^47^. Odors may have different stimulus residual times that can be perceived by animals depending on the type of sources, such as passing animals, urine, or feces. Therefore, it may be important to set a temporal scale that considers the type of odor sources in assessing species interactions. Our results suggest that factors affecting the assessment of species interactions (i.e., the availability of prey species, weather conditions, and odors) may differ depending on the temporal scales. Therefore, it is important to assess the temporal niche partitioning on temporal scales within which species interactions can be detected.

The temporal co-occurrence analyses in units of one night performed in this study did not detect co-occurrence nor partitioning between red foxes and Japanese martens (Tables 1 and 2), even though their diel activities highly overlapped (Fig. 3). This indicated that the overlaps of diel activity patterns were not always consistent to the spatio-temporal niche overlaps. This study suggested that the temporal co-occurrence analysis may be an alternative method to assess the temporal interactions between competitive species that cannot be detected by the overlaps of diel activity patterns. The temporal co-occurrence analyses might have methodological advantages, owing to their use of presence-absence data. For example, presence-absence data are typically available in large amounts of data, compared to those of other methods, such as the MRPP. Indeed, Tattersall et al.^69^ indicated that the assessment of the temporal niche partitioning at fine temporal scales required large amounts of the detection data and applied presence-absence data for the assessment. Further, the spatial co-occurrence analysis has been often used in studies focused on spatial niche partitioning and its associated factors (e.g., weather conditions or geo-environmental gradients^44^). Therefore, the knowledge and methodology applied in spatial co-occurrence studies may be applicable to temporal assessments. However, the application of this approach to temporal data has not been verified by this study only, and reliability needs to be evaluated with various data. Moreover, the conditions for application of this approach will require further study.

In this study, we also assessed the before/after occurrence differences in a species pair (e.g., red fox (before)—raccoon dog (after), and raccoon dog—red fox) in the MRPP and 15 h-MRPP analyses; the results differed depending on the species-occurrence order (Figs. 4 and 5). However, many previous studies disregarded the effects of this order^33,36,37,70^ (but also see Paúl et al.^38^). This study suggested the necessity of the time-to-encounter analysis with the species-occurrence order replacement, if the competitive dominances among focal species are not clear. Although the 15 h-MRPP analyses indicated the possibility of behavioral avoidances by Japanese martens to the lager species, there were weak evidences in the statistical assessments, possibly due to the small sample sizes (Fig. 5). Niedballa et al.^32^ indicated that more than 100 samples per species are needed to perform the time-to-encounter analysis with statistical validations. It will be important to use 15 h-MRPP based on a larger sample size in the future. However, our findings suggest that the time-to-encounter analysis with a fine temporal scale dataset may be a powerful tool to assess spatio-temporal partitioning between nocturnal carnivores, when the sample sizes of both species are adequate.

In this study, there may be some concerns about the placement of camera trap sites. Although the spatial similarity of the camera trap results was low (Supplementary Figs. S1 and S2), the sampling design of placing the cameras on the connected forest roads may have created a bias that the detection events were not independent. Since the number of detections is important for the evaluation of niche partitioning, camera traps were placed on forest roads where many target species were photographed in the study area to ensure the number of detections in this study. However, evaluating the spatio-temporal niche partitioning approach would require further consideration of the sampling design. In addition, the different survey periods of active camera traps at each site may also create a bias against the results of the co-occurrence analysis and MRPP. Although no problems were detected during the preliminary analysis in this study, it may be necessary to note the uneven active periods of the camera trapping surveys in some cases.

We evaluated the temporal and spatio-temporal niche partitioning between carnivores on a temporal scale within which species interactions can be detected by using multiple analytical methods with different uses of temporal data. The results of the assessment of the niche partitioning differed depending on the analytical methods, and therefore the choice of analytical approach is important for understanding the mechanisms of species coexistence based on their temporal and spatio-temporal niche partitioning. Our results suggested that only using the coefficient of temporal overlap to determine the temporal niche partitioning between species is insufficient for assessing species interactions. Therefore, we recommend the use of multiple methods by temporal co-occurrence analysis and/or time-to-encounter analysis based on spatio-temporal partitioning in addition to the evaluation by the temporal overlap. Our results also suggest that the factors affecting species interactions may differ depending on the temporal scales. It is important to assess temporal and spatio-temporal niche partitioning on a detailed temporal scale within which species interactions can be detected by using the time-to-encounter analysis with an upper limit on the time-to-encounter data. Adopting this approach would provide a better understanding of the mechanisms determining species coexistence.

## Supplementary Information

Supplementary Information.

## Data Availability

The datasets generated during and/or analyzed during the current study are available from the corresponding author on reasonable request.

## References

[1] Gause GF (1934). Experimental analysis of Vito Volterra’s mathematical theory of the struggle for existence. Science.

[2] Amarasekare P (2003). Competitive coexistence in spatially structured environments: A synthesis. Ecol. Lett..

[3] HilleRisLambers J, Adler PB, Harpole WS, Levine JM, Mayfield MM (2012). Rethinking community assembly through the lens of coexistence theory. Annu. Rev. Ecol. Evol. Syst..

[4] Wisz MS (2013). The role of biotic interactions in shaping distributions and realised assemblages of species: Implications for species distribution modelling. Biol. Rev..

[5] Frey S, Fisher JT, Burton AC, Volpe JP (2017). Investigating animal activity patterns and temporal niche partitioning using camera-trap data: Challenges and opportunities. Remote Sens. Ecol. Conserv..

[6] Davis CL (2018). Ecological correlates of the spatial co-occurrence of sympatric mammalian carnivores worldwide. Ecol. Lett..

[7] Durant SM (1998). Competition refuges and coexistence: An example from Serengeti carnivores. J. Anim. Ecol..

[8] Fedriani JM, Fuller TK, Sauvajot RM, York EC (2000). Competition and intraguild predation among three sympatric carnivores. Oecologia.

[9] Kamler JF, Ballard WB, Gilliland RL, Mote K (2003). Spatial relationships between swift foxes and coyotes in northwestern Texas. Can. J. Zool..

[10] Vanak AT (2013). Moving to stay in place: Behavioral mechanisms for coexistence of African large carnivores. Ecology.

[11] Donadio E, Buskirk SW (2006). Diet, morphology, and interspecific killing in carnivora. Am. Nat..

[12] Tsunoda H (2017). Food niche segregation between sympatric golden jackals and red foxes in central Bulgaria. J. Zool..

[13] Palomares F, Caro TM (1999). Interspecific killing among mammalian carnivores. Am. Nat..

[14] Linnell JDC, Strand O (2000). Interference interactions, co-existence and conservation of mammalian carnivores. Divers. Distrib..

[15] Kamler JF, Stenkewitz U, Klare U, Jacobsen NF, MacDonald DW (2012). Resource partitioning among cape foxes, bat-eared foxes, and black-backed jackals in South Africa. J. Wildl. Manag..

[16] Di Bitetti MS, Di Blanco YE, Pereira JA, Paviolo A, Pírez IJ (2009). Time Partitioning favors the coexistence of sympatric crab-eating foxes (*Cerdocyon thous*) and Pampas Foxes (*Lycalopex gymnocercus*). J. Mammal..

[17] Lesmeister DB, Nielsen CK, Schauber EM, Hellgren EC (2015). Spatial and temporal structure of a mesocarnivore guild in Midwestern North America. Wildl. Monogr..

[18] Di Bitetti MS, De Angelo CD, Di Blanco YE, Paviolo A (2010). Niche partitioning and species coexistence in a Neotropical felid assemblage. Acta Oecologica.

[19] Monterroso P, Alves PC, Ferreras P (2014). Plasticity in circadian activity patterns of mesocarnivores in southwestern Europe: Implications for species coexistence. Behav. Ecol. Sociobiol..

[20] Tsunoda H, Ito K, Peeva S, Raichev E, Kaneko Y (2018). Spatial and temporal separation between the golden jackal and three sympatric carnivores in a human-modified landscape in central Bulgaria. Zool. Ecol..

[21] Tsunoda H (2020). Spatio-temporal partitioning facilitates mesocarnivore sympatry in the Stara Planina Mountains, Bulgaria. Zoology.

[22] Ramesh T, Kalle R, Sankar K, Qureshi Q (2012). Spatio-temporal partitioning among large carnivores in relation to major prey species in Western Ghats. J. Zool..

[23] Gómez-Ortiz Y, Monroy-Vilchis O, Castro-Arellano I (2019). Temporal coexistence in a carnivore assemblage from central Mexico: Temporal-domain dependence. Mammal Res..

[24] Ridout MS, Linkie M (2009). Estimating overlap of daily activity patterns from camera trap data. J. Agric. Biol. Environ. Stat..

[25] Meredith, M. & Ridout, M. Overlap: Estimates of coefficient of overlapping for animal activity patterns. https://cran.r-project.org/web/packages/overlaphttps://cran.r-project.org/web/packages/overlap/index.html (2018).

[26] Marinho PH, Fonseca CR, Sarmento P, Fonseca C, Venticinque EM (2020). Temporal niche overlap among mesocarnivores in a Caatinga dry forest. Eur. J. Wildl. Res..

[27] Vilella M, Ferrandiz-Rovira M, Sayol F (2020). Coexistence of predators in time: Effects of season and prey availability on species activity within a Mediterranean carnivore guild. Ecol. Evol..

[28] Zhao G (2020). Spatio-temporal coexistence of sympatric mesocarnivores with a single apex carnivore in a fine-scale landscape. Glob. Ecol. Conserv..

[29] Farmer MJ, Allen ML, Olson ER, Van Stappen J, Van Deelen TR (2021). Agonistic interactions and island biogeography as drivers of carnivore spatial and temporal activity at multiple scales. Can. J. Zool..

[30] Watabe R, Saito MU (2021). Diel activity patterns of three sympatric medium-sized carnivores during winter and spring in a heavy snowfall area in northeastern Japan. Mammal Study.

[31] Lashley MA (2018). Estimating wildlife activity curves: comparison of methods and sample size. Sci. Rep..

[32] Niedballa J, Wilting A, Sollmann R, Hofer H, Courtiol A (2019). Assessing analytical methods for detecting spatiotemporal interactions between species from camera trapping data. Remote Sens. Ecol. Conserv..

[33] Karanth KU (2017). Spatio-temporal interactions facilitate large carnivore sympatry across a resource gradient. Proc. R. Soc. B Biol. Sci..

[34] Cusack JJ (2017). Revealing kleptoparasitic and predatory tendencies in an African mammal community using camera traps: A comparison of spatiotemporal approaches. Oikos.

[35] Balme G (2019). Big cats at large: density, structure, and spatio-temporal patterns of a leopard population free of anthropogenic mortality. Popul. Ecol..

[36] Li Z (2019). Coexistence of two sympatric flagship carnivores in the human-dominated forest landscapes of Northeast Asia. Landsc. Ecol..

[37] Lahkar D, Ahmed MF, Begum RH, Das SK, Harihar A (2021). Inferring patterns of sympatry among large carnivores in Manas National Park: A prey-rich habitat influenced by anthropogenic disturbances. Anim. Conserv..

[38] Paúl MJ, Layna JF, Monterroso P, Álvares F (2020). Resource partitioning of sympatric African Wolves (*Canis lupaster*) and side-striped jackals (*Canis adustus*) in an arid environment from West Africa. Diversity.

[39] Prat-Guitart M, Onorato DP, Hines JE, Oli MK (2020). Spatiotemporal pattern of interactions between an apex predator and sympatric species. J. Mammal..

[40] Stone L, Roberts A (1990). The checkerboard score and species distributions. Oecologia.

[41] Griffith DM, Veech JA, Marsh CJ (2016). Cooccur: Probabilistic species co-occurrence analysis in r. J. Stat. Softw..

[42] Noor A, Mir ZR, Veeraswami GG, Habib B (2017). Activity patterns and spatial co-occurrence of sympatric mammals in the moist temperate forest of the Kashmir Himalaya, India. Folia Zool..

[43] de Satgé J, Teichman K, Cristescu B (2017). Competition and coexistence in a small carnivore guild. Oecologia.

[44] Kass JM, Tingley MW, Tetsuya T, Koike F (2020). Co-occurrence of invasive and native carnivorans affects occupancy patterns across environmental gradients. Biol. Invasions.

[45] Louppe V, Herrel A, Pisanu B, Grouard S, Veron G (2020). Assessing occupancy and activity of two invasive carnivores in two Caribbean islands: implications for insular ecosystems. J. Zool..

[46] Proulx G, Harrison DJ, Fuller AK, Proulx G (2005). World distribution and status of the genus Martes in 20. Martens and Fishers (Martes) in Human-Altered Environments.

[47] Ohdachi SD, Ishibashi Y, Iwasa M, Fukuki D, Saitoh T (2015). The Wild Mammals of Japan.

[48] Kauhala, K. & Saeki, M. Nyctereutes procyonoides. The IUCN Red List of Threatened Species. https://www.iucnredlist.org/species/14925/85658776 (2016).

[49] Yamamoto Y (1994). Comparative analyses on food habits of Japanese marten, red fox, badger and raccoon dog in the Mt. Nyugasa, Nagano Prefecture, Japan. Nat. Environ. Sci. Res..

[50] Hisano M (2017). A comparison of visual and genetic techniques for identifying Japanese marten scats enabling diet examination in relation to seasonal food availability in a sub-alpine area of Japan. Zool. Sci..

[51] Lindstrom ER, Brainerd SM, Helldin JO, Overskaug K (1995). Pine marten-red fox interactions: A case of intraguild predation?. Ann. Zool. Fenn..

[52] Waggershauser CN, Ruffino L, Kortland K, Lambin X (2021). Lethal interactions among forest-grouse predators are numerous, motivated by hunger and carcasses, and their impacts determined by the demographic value of the victims. Ecol. Evol..

[53] Watabe R, Saito MU, Enari HS, Enari H (2020). Mammalian fauna of the Kaminagawa Experimental Forest of Yamagata University detected by camera traps. Tohoku J. For. Sci..

[54] Hofmeester TR, Rowcliffe JM, Jansen PA (2017). A simple method for estimating the effective detection distance of camera traps. Remote Sens. Ecol. Conserv..

[55] Di Bitetti MS, Paviolo A, De Angelo C (2014). Camera trap photographic rates on roads vs. off roads: Location does matter. Mastozoología Neotrop..

[56] Borcard D, Legendre P (2012). Is the Mantel correlogram powerful enough to be useful in ecological analysis? A simulation study. Ecology.

[57] Oksanen, J. *et al.* Vegan: community ecology package. https://cran.r-project.org/web/packages/veganhttps://cran.r-project.org/web/packages/vegan/index.html (2019).

[58] R Core Team. R: a language environment for statistical computing. r foundation for statistical computing, Vienna, Austria. https://www.r-project.org/https://www.r-project.org/ (2021).

[59] Linkie M, Ridout MS (2011). Assessing tiger-prey interactions in Sumatran rainforests. J. Zool..

[60] Watabe R, Saito MU (2021). Effects of vehicle-passing frequency on forest roads on the activity patterns of carnivores. Landsc. Ecol. Eng..

[61] Furukawa, G. genkiFurukawa/rSetDayNightAttr documentation. https://rdrr.io/github/genkiFurukawa/rSetDayNightAhttps://rdrr.io/github/genkiFurukawa/rSetDayNightAttr/ (2019).

[62] Mielke PW, Berry KJ, Johnson ES (1976). Multi-response permutation procedures for a priori classifications. Commun. Stat. Theory Methods.

[63] Kronfeld-Schor N, Dayan T (2003). Partitioning of time as an ecological resource. Annu. Rev. Ecol. Evol. Syst..

[64] Monterroso P, Alves PC, Ferreras P (2013). Catch me if you can: Diel activity patterns of mammalian prey and predators. Ethology.

[65] Hendrichsen DK, Tyler NJC (2014). How the timing of weather events influences early development in a large mammal. Ecology.

[66] Herfindal I (2017). Weather affects temporal niche partitioning between moose and livestock. Wildlife Biol..

[67] Haswell PM, Jones KA, Kusak J, Hayward MW (2018). Fear, foraging and olfaction: How mesopredators avoid costly interactions with apex predators. Oecologia.

[68] Barrull J (2014). Factors and mechanisms that explain coexistence in a Mediterranean carnivore assemblage: An integrated study based on camera trapping and diet. Mamm. Biol..

[69] Tattersall ER, Burgar JM, Fisher JT, Burton AC (2020). Boreal predator co-occurrences reveal shared use of seismic lines in a working landscape. Ecol. Evol..

[70] Moll RJ (2018). Humans and urban development mediate the sympatry of competing carnivores. Urban Ecosyst..

[71] McCreadie JW, Bedwell CR (2013). Patterns of co-occurrence of stream insects and an examination of a causal mechanism: Ecological checkerboard or habitat checkerboard?. Insect Conserv. Divers..

