# Supplementary material for: Evaluating the temporal and spatio-temporal niche partitioning between carnivores by different analytical method in northeastern Japan
Source: Sci Rep. 2022 Jul 14;12:11987. doi: 10.1038/s41598-022-16020-w (PMC9283404; doi:10.1038/s41598-022-16020-w)
Supplement: Supplementary file 1 — Supplementary Information. [file 41598_2022_16020_MOESM1_ESM.pdf]

Supplementary Table S1. Number of camera-trap sites and survey duration.

Survey duration	Number of camera-trap sites
May1–Oct 31, 2019, May 1–Oct 31, 2020	3
May 1–Oct 31, 2019, Aug 24–Oct 31, 2020	1
May 1–Oct 31, 2019	3
May 1–Oct 31, 2020	1
Aug 19–Oct 31, 2019, Aug 24–Oct 31, 2020	1
Aug 19–Oct 31, 2019	3
Aug 24–Oct 31, 2020	6

Supplementary Table S2. Number of detection samples for each survey duration in the time-to-encounter analysis.

Species	Survey duration			
	May 1 -Oct 31, 2019	Aug 19-Oct 31, 2019	May 1-Oct 31, 2020	Aug 24-Oct 31, 2020
Red fox	375	77	108	145
Raccoon dog	270	128	120	217
Japanese marten	118	40	78	53

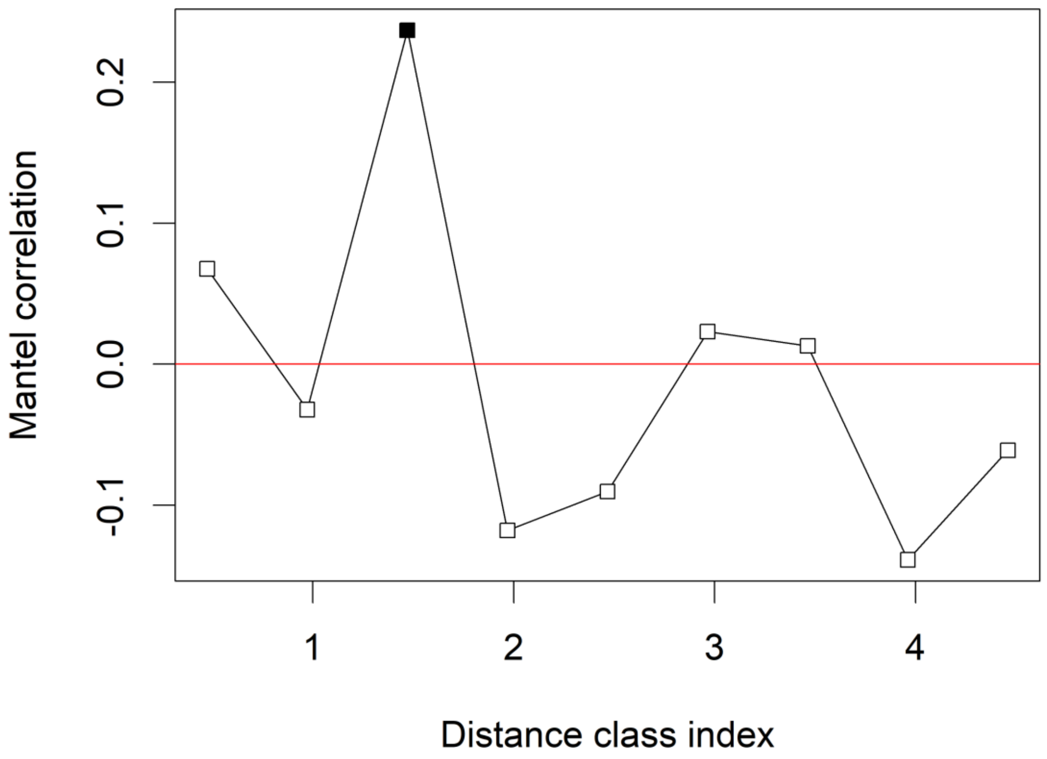

Supplementary Figure S1. Mantel's correlogram to assess spatial autocorrelations for target species. The solid plot represents statistical significance on the Mantel's correlation index.

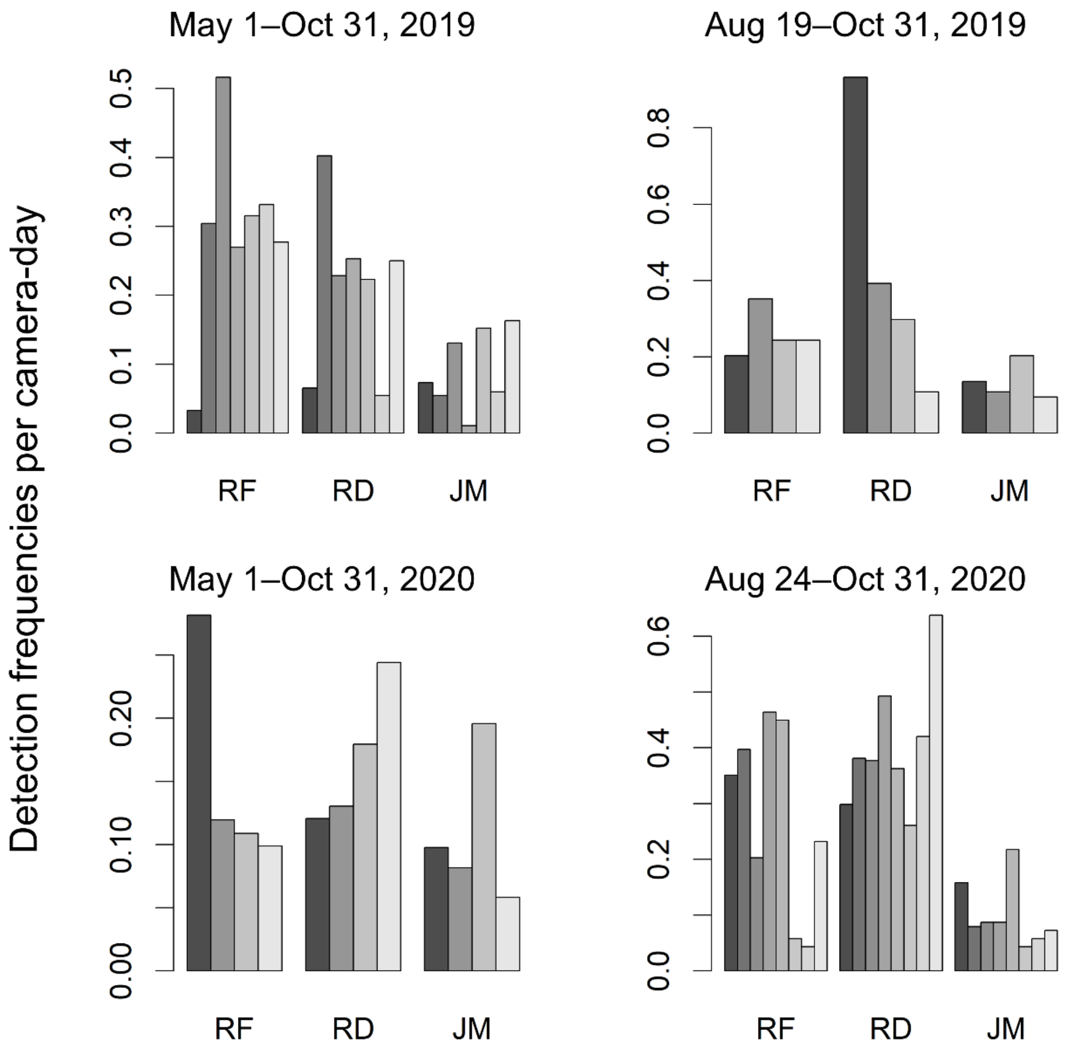

Supplementary Figure S2. Relative detection frequencies for each camera-trap site in the same survey period. The upper of the plot represents the survey period with the camera-traps working continuously. The barplot represents the detection frequencies for each camera-trap site in the period, with the closest cameras next to each other. RF, RD, and JM represents the red fox, raccoon dog, and Japanese marten.
